# Optimization of the Use of Generic Medications in Oncology: Improving Safety and Therapeutic Quality

**DOI:** 10.3390/jcm14217543

**Published:** 2025-10-24

**Authors:** Diego Gómez Abreo, Daniel F. Alarcón Cano, Fernando Ayala, Nelson Belalcázar Carvajal, Marie Claire Berrouet Mejía, Oscar Beltrán, Carlos Alberto Calderón-Ospina, Hugo Castro-Salguero, Diana Marcela Escobar Cárdenas, Mauricio Lema-Medina, Juan Ignacio Marín Zuluaga, Pilar Milla Bernabé, Rafael E. Niño Velasco, Ruth Osorio Estévez, Jorge Mario Ortiz, Leonardo J. Rojas-Melo, Marcela Urrego, Carlos Vargas, Andrés F. Zuluaga

**Affiliations:** 1 International Hospital of Colombia, Bucaramanga 681011, Colombia; 2 Society for the Fight Against Cancer, Portoviejo 130106, Ecuador; alarcon.daniel.md@gmail.com; 3 University Hospital Fundación SantaFé de Bogotá, Bogotá 110211, Colombia; ferchoayal@hotmail.com (F.A.); carlovic17@gmail.com (C.V.); 4 The Board of Oncologists of the West S.A.S and the Consultation Area, Bogotá 110131, Colombia; nbelalcazar@gmail.com; 5 Department of Clinical Toxicology, School of Medicine, Faculty of Health Sciences, CES University, Medellín 050015, Colombia; mcberrouet@hotmail.com; 6 General Hospital of Medellín, Medellín 050015, Colombia; 7 The Transplant Group of the Cardio Infantil Foundation/La Cardio, Bogotá 110131, Colombia; obeltran2@mac.com; 8 The Gastroenterology Program at the University of Rosario, Bogotá, 110131, Colombia; 9 Research Group in Applied Biomedical Sciences (UR Biomed), School of Medicine and Health Sciences, Universidad del Rosario, Bogota 111221, Colombia; carlos.calderon@urosario.edu.co; 10 Medical Group Ángeles, Guatemala City 01001, Guatemala; hugoraulcastro@hotmail.com; 11 Valle de Lili Foundation, Cali 760044, Colombia; dianamarcelaescobar@hotmail.es; 12 Astorga Oncology Clinic, Medellín 050022, Colombia; mauriciolena@yahoo.com; 13 Pablo Tobón Hospital, Medellín 050010, Colombia; marinji@hotmail.com; 14 Guillermo Almenara Irigoyen Hospital, Lima 15033, Peru; pilardency@gmail.com; 15 Cancerology Clinic of Norte de Santander, Cúcuta 540006, Colombia; rafaelninovelasco@gmail.com (R.E.N.V.); draruthosorio@gmail.com (R.O.E.); 16 Clinical Hepatology, IMAT Oncomedica (IMAT S.A.S), Montería 230002, Colombia; drjortizpatron@gmail.com; 17 San Ignacio University Hospital, Bogotá 110221, Colombia; leonardorojasmd@yahoo.com; 18 CTIC Center for Cancer Treatment and Research, Bogotá 110221, Colombia; 19 Sebastián de Belalcázar Clinic, Cali 760044, Colombia; murregom@gmail.com; 20 Integrated Laboratory of Specialized Medicine (LIME), Faculty of Medicine, University of Antioquia, Medellín 050010, Colombia; andres.zuluaga@udea.edu.co

**Keywords:** generic drugs, oncology, bioequivalence, regulation, safety and efficacy, Delphi consensus

## Abstract

**Introduction**: Oncological diseases are one of the leading causes of mortality worldwide, with the high cost of therapies representing a critical barrier for health systems. Generic drugs have emerged as an alternative to reduce costs and improve access; however, their quality, safety, and efficacy remain a subject of regulatory and clinical debate. This issue is particularly sensitive in oncology, where generics often involve cytotoxic agents, narrow therapeutic indices, and complex formulations, all of which amplify the risks of therapeutic interchangeability. **Materials and Methods**: A multidisciplinary team composed of 19 experts in oncology, hepatology, gastroenterology, toxicology, endocrinology, and pharmacology was convened based on established academic contributions, clinical expertise, and participation in regulatory or guideline development. Evidence was synthesized through a non-systematic narrative review of PubMed, Embase, and regional databases. Consensus recommendations were developed using a two-round Delphi process, with agreement defined as ≥75%. **Results**: The Delphi panel produced six key recommendations: (1) stricter requirements for bioequivalence and bioavailability, tailored to oncology; (2) strengthened pharmacovigilance and real-world monitoring; (3) standardized protocols for therapeutic interchangeability, particularly for narrow therapeutic index agents; (4) active physician involvement in formulary decision-making; (5) harmonized regional regulatory frameworks, informed by FDA and EMA standards; and (6) expanded research on oncology-specific pharmacokinetic markers. While safety concerns dominated discussions, experts also acknowledged the potential of generics to reduce costs, improve equity, and enhance the sustainability of oncology care. **Conclusions**: The findings underscore the need for oncology-specific regulatory frameworks that extend beyond conventional bioequivalence standards. A balance is required: cost savings and equity gains offered by generics must be matched with robust safety mechanisms, regulatory harmonization, and physician-led oversight. Future research should expand expert representation, integrate real-world data, and address biosimilars in dedicated analyses to ensure safe and equitable integration of non-innovator therapies in cancer care.

## 1. Introduction

Oncological diseases represent one of the leading causes of mortality worldwide [1]. The high cost of treatments constitutes a significant barrier for many health systems [2,3]. Generic and biosimilar medications have emerged as essential strategies to reduce costs and improve access to effective therapies, particularly in oncology, where treatment expenditures are rapidly increasing [4,5].

Generics are defined as pharmaceutical products designed to be interchangeable with reference (innovator) drugs, with demonstrated bioequivalence in composition, potency, dosage form, route of administration, and pharmacological activity [2,6]. Their use is steadily growing, with oncology generics expected to reach sustained market expansion between 2020 and 2024, generating substantial cost savings [7,8,9,10].

However, the adoption of oncology generics raises unique challenges given their narrow therapeutic index, cytotoxic nature, and complex formulations, where even minor variations in absorption or metabolism may compromise therapeutic efficacy and patient survival [11].

Despite regulatory frameworks requiring demonstration of bioequivalence, oncology generics are often approved without oncology-specific clinical studies, contributing to persistent uncertainty regarding their real-world effectiveness and safety compared with innovator products. Strengthened regulatory oversight, harmonized international standards, and robust post-marketing pharmacovigilance are therefore critical to ensure safe integration into cancer care [12]. This study aims to develop consensus-based recommendations for the optimal use of generic and biosimilar medications in oncology. Using a Delphi methodology, we integrate expert perspectives with regulatory analysis to provide practical guidance for improving safety, efficacy, and access in real-world oncology practice.

### 1.1. Regulatory Context

Regulatory frameworks for generics in Latin America remain highly heterogeneous, with important implications for oncology (Table S1) [2]. In 2010, only half of the countries in the region required bioequivalence testing for market approval [13]. Although progress has been made, definitions and requirements for generics continue to differ substantially across countries [13,14,15]. This regulatory fragmentation limits the consistency, availability, and trust in oncology generics.

International experiences highlight the risks of insufficient oversight. In 2022, the European Medicines Agency (EMA) suspended more than 100 generics due to defective bioequivalence studies [16]. More recently, the International Council for Harmonization (ICH) published guidance for both complex and non-complex dosage forms [17], reinforcing the need for rigorous, transparent, and harmonized standards globally. For oncology, where treatment failure can directly impact survival, regulatory variability poses a particularly critical challenge.

### 1.2. Bioequivalence and Its Limitations in Oncology

Bioequivalence is established when the rate and extent of absorption of a generic are not significantly different from its innovator product [18], usually requiring that the 90% confidence interval for pharmacokinetic parameters (AUC and Cmax) falls within 80–125% [19,20]. These studies are often conducted in healthy volunteers [21], which raises concerns about their relevance for oncology populations with altered physiology.

The U.S. Food and Drug Administration (FDA) and EMA accept therapeutic equivalence even when differences exist in formulation characteristics such as excipients, release profiles, or packaging [22]. While acceptable for many drug classes, these variations may have disproportionate clinical consequences in oncology, particularly for agents with a narrow therapeutic index or cytotoxic activity.

### 1.3. Factors Influencing Safety and Efficacy

**Figure 1:**
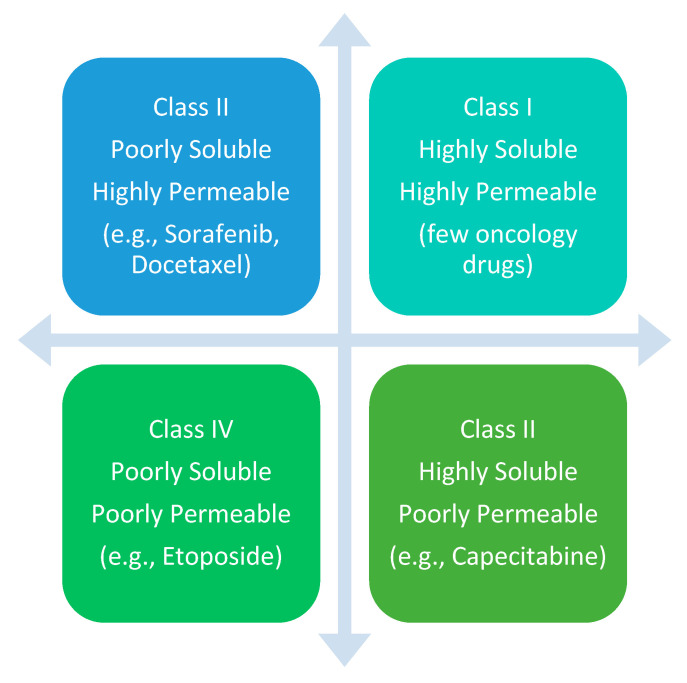
Biopharmaceutical Classification System (BCS).

Molecule Class: Many oncology drugs belong to Biopharmaceutical Classification System (BCS) class II or IV, characterized by low solubility and/or permeability (Figure 1) [23]. For such drugs, bioequivalence is harder to demonstrate, and small formulation differences may alter clinical activity. Moreover, relying solely on solubility and permeability may ignore patient-specific factors—such as disease state and gastrointestinal function—that influence drug absorption and metabolism in cancer populations.

Excipients and Impurities: Variability in excipients or the presence of impurities can alter solubility, absorption, or toxicity [17]. For complex molecules such as tyrosine kinase inhibitors, inappropriate excipient use or failure to replicate sophisticated formulation technologies (e.g., amorphous solid dispersions in sorafenib) may compromise efficacy and safety [24,25,26].

Stability and Packaging: In tropical regions, inadequate packaging and storage conditions can accelerate drug degradation, reducing potency and therapeutic effect [27].

Polymorphisms and manufacturing quality: Variability in crystalline forms [28,29], substandard active ingredient content [30], or insufficient process controls [27,31,32,33,34] can affect bioavailability and therapeutic outcomes.

Together, these factors underscore why oncology generics require stricter scrutiny than standard generics, including more rigorous evaluation of formulation, stability, and manufacturing consistency.

### 1.4. Evidence Gaps and Need for Surveillance

Despite the availability of generics, major evidence gaps remain. Few real-world studies compare oncology generics and innovator drugs in terms of survival, toxicity, or long-term outcomes. Stability under local storage conditions, especially in Latin America, is poorly documented, and bioequivalence studies in healthy volunteers may not capture variability in oncology patients. Additional challenges include the absence of harmonized regional frameworks, limited data on complex molecules, and insufficient transparency in quality control.

Strengthening pharmacovigilance systems, mandating oncology-specific post-marketing studies, and harmonizing international regulatory standards are essential to address these gaps and ensure the safe adoption of generics in oncology.

This study seeks to develop consensus-based recommendations to optimize the use of generic and biosimilar oncology medications. Using a Delphi methodology, we integrate expert perspectives with regulatory analysis to propose practical strategies for ensuring safety, efficacy, and accessibility in real-world cancer care.

## 2. Materials and Methods

### 2.1. Formation of the Working Group

A working group was formed consisting of 19 participants, including hepatologists, pharmacologists, oncologists, gastroenterologists, endocrinologists, and toxicologists. Selection criteria included at least 10 years of clinical or academic experience in oncology or pharmacology, prior publications or leadership roles in relevant fields, and representation from both academic and hospital-based institutions. Geographic diversity was sought across Latin America to capture regional heterogeneity, although participation was limited to this region.

The panel was supported by an independent methodology expert, whose role was to ensure adherence to the Delphi process, supervise survey design and statistical analysis, and maintain neutrality. Importantly, the methodology expert did not participate in content voting or drafting recommendations, thereby preserving independence. A leader (D.G.) coordinated the process, structured the agenda, and integrated panel feedback, but voting remained fully anonymous and independent.

### 2.2. Literature Review

The evidence base was informed by a non-systematic (narrative) literature review. Searches were conducted in PubMed, Embase, and SciELO covering 2000–2023. Inclusion criteria were studies addressing generic or biosimilar oncology drugs, bioequivalence, pharmacovigilance, or regulation. Exclusion criteria included case reports and non-peer-reviewed sources. This narrative approach was chosen to provide a broad overview of regulatory and clinical issues rather than a structured evidence synthesis. Six panelists presented focused reviews summarizing regulatory frameworks, clinical outcomes, and pharmacological aspects as a prelude to the Delphi process.

### 2.3. Development of Recommendations

Recommendations were refined through a Delphi process. Two Delphi rounds were conducted using an online survey tool. Each statement was rated on a Likert scale from 1 (“strongly disagree/extremely irrelevant”) to 10 (“strongly agree/extremely relevant”). Consensus was defined as a median score > 8, following prior Delphi methodology in clinical guideline development [35,36]. Mean scores were chosen over median values to summarize expert ratings. While the median is often preferred in Delphi studies for its robustness against outliers, the mean offers complementary advantages in contexts where the full distribution of responses is relevant. Specifically, the mean provides a more sensitive measure of central tendency that reflects even subtle shifts in group opinion. This is particularly valuable when response distributions are relatively homogeneous and not unduly skewed, as it allows for greater discrimination between closely ranked items.

Consensus stability was assessed by comparing response distributions across rounds. Items with persistent variability were rephrased and revoted. Outliers and minority positions were documented and discussed during plenary sessions to ensure transparency. Divergent views that did not reach consensus were retained as “non-consensus statements” and are reported separately.

Drafting was performed by two subgroups, and their outputs were merged into unified recommendation statements before voting.

### 2.4. Open Debate

An in-person meeting was held after the second Delphi round. This forum allowed critical appraisal, discussion of unresolved items, and final confirmation of consensus recommendations. Disagreements were openly debated, but final votes were anonymous, ensuring that discussion did not bias responses.

### 2.5. Independence Assurance

The process was supported logistically by Bayer, but the sponsor had no role in topic selection, survey design, data analysis, or drafting of recommendations. Oversight of methodology and publication was conducted by the independent methodology expert and an external medical writer.

The rationale for hosting this consensus independently rather than under the auspices of a medical society was primarily logistical: rapid coordination, regional inclusivity, and avoidance of the administrative delays often associated with formal society endorsement. This independent approach facilitated broader participation across institutions, though it may limit the formal authority of the recommendations; for this reason, transparency in reporting methodology and independence was prioritized to reinforce credibility.

## 3. Results

### 3.1. Delphi Panel Consensus

Table 1 presents the voting scores from the Delphi panel across key domains, including perceptions of innovator versus generic drug profiles, the clinical relevance of bioavailability in oncology, and the assessment of bioequivalence and clinical outcomes. These scores represent expert consensus rather than direct clinical evidence. The Delphi process was conducted in two sequential phases: an open-ended question phase, followed by a phase of structured recommendations.

**Figure 2:**
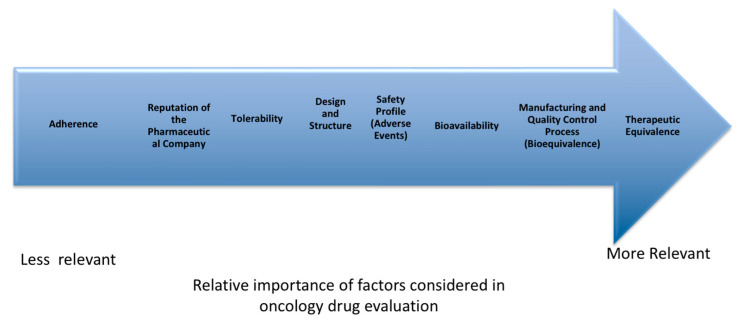
Critical differences between generic and innovator molecules.

From the open-ended questions, the critical differences between generic and innovator molecules that may have a potential impact on clinical response were illustrated in Figure 2.

### 3.2. Evidence from Peer-Reviewed Studies

In contrast to the panel’s structured ratings, published studies provide empirical evidence of clinical differences between innovator and generic oncology drugs. Representative case examples are consolidated in Table 2. For instance, generic imatinib in chronic myeloid leukemia has been associated with higher therapeutic failure rates [38,39] and increased toxicity [40,41,42,43], while generic abiraterone and aromatase inhibitors in prostate cancer showed reduced PSA suppression, a key biochemical marker of efficacy. Among solid tumor treatments, generic docetaxel revealed substandard active pharmaceutical ingredient (API) content [10 versions met expected API levels (90–110%), 21 were below 90%, and 11 below 80%] [34] and contamination [1–3% rate] [21], while generic cisplatin was associated with significantly higher renal failure rates (21% vs. 9%; *p* < 0.001; 95% CI for difference: 7.2–16.8%) [44].

## 4. Discussion

### 4.1. Importance of Rigorous Regulation and Control in the Authorization of Generic Medications

To ensure that generic medications meet the same standards of quality, safety, and efficacy as innovative products, it is essential that regulatory agencies, such as INVIMA, implement stricter measures for their approval. Bioequivalence studies should be complemented with more rigorous research that specifically addresses the needs of cancer patients and other complex conditions. Furthermore, regulatory oversight is required for medications approved by international agencies such as the FDA and EMA, to ensure that the pursuit of lower-cost alternatives does not compromise patient health.

### 4.2. Active Participation of Physicians in Treatment Selection

Beyond regulatory oversight, the role of physicians is pivotal in safeguarding treatment quality when generics are introduced. Evidence shows that clinician involvement in formulary decision-making improves the alignment of institutional drug choices with patient needs, enhances adherence to evidence-based practices, and reduces inappropriate substitutions that may compromise safety [46,47,48]. In oncology, where treatment precision is critical, the physician’s role extends beyond prescribing to active participation in pharmacy committees and policy discussions.

Studies have demonstrated that when oncologists and multidisciplinary teams are directly involved in formulary decisions, patient outcomes improve due to more tailored drug selection, closer pharmacovigilance, and faster identification of adverse trends associated with generic substitutions [46,47,48]. This participatory approach also mitigates undue influence from economic or industrial pressures, ensuring decisions remain focused on clinical benefit rather than cost alone. Importantly, informed physician engagement is essential in monitoring therapeutic switches between innovator and generic products, as these transitions can have significant implications for patient response and toxicity profiles.

### 4.3. Ensuring Adequate Access to Quality Medications

Although generic medications play a vital role in improving access and reducing costs, their introduction to the market must follow the same standards of post-marketing surveillance applied to innovative products. Continuous monitoring of side effects and adverse events is required to allow timely regulatory adjustments and safeguard patient safety. Any reduction in direct treatment costs should not come at the expense of therapeutic quality, particularly in oncology, where compromised efficacy can result in serious complications, relapses, progression, or adverse effects that impact long-term health or even increase mortality.

### 4.4. The SORDOS Strategy

The SORDOS strategy represents a conceptual framework aimed at systematizing information, organizing professional networks, fostering collective responses, disseminating evidence, advocating to decision-makers, and synthesizing knowledge for the broader community. While its value lies in providing a structured roadmap to improve access to high-quality generics, it remains a proposal that requires further validation.

A critical limitation is the absence of pilot testing or real-world implementation data. Without empirical evidence, it is difficult to determine its feasibility, scalability, or sustainability across diverse health systems, particularly in low- and middle-income countries where regulatory and resource constraints are more pronounced. Comparable initiatives—such as WHO’s “Good Governance for Medicines” framework or regional pharmacovigilance networks in Latin America—demonstrate that structured, multi-stakeholder approaches can improve transparency and drug safety, but they also highlight challenges in coordination, funding, and political commitment [49]. Lessons from these models suggest that SORDOS could benefit from phased pilot studies in selected oncology centers, accompanied by measurable outcomes such as timeliness of adverse event reporting, physician engagement in formulary decisions, and regulatory responsiveness.

Moreover, the strategy’s success would depend on meaningful physician participation. Evidence from formulary management and antimicrobial stewardship programs shows that when clinicians are directly engaged, treatment appropriateness improves and adverse outcomes are reduced [50,51]. Embedding SORDOS within existing institutional committees, rather than as a parallel structure, may increase acceptance and sustainability.

### 4.5. Role of Real-World Evidence

Assessing long-term outcomes such as overall survival (OS) in oncology is challenging, given the complexity of treatment pathways and multiple lines of therapy. In real-world settings, progression-free survival (PFS) stratified by cancer type and stage may serve as a more practical endpoint. However, in Latin America and other regions, variability in treatment access complicates direct comparisons. Ideally, prospective measurement of pharmacokinetic markers—such as serum drug levels, trough concentrations, and validated biomarkers (e.g., PSA in prostate cancer)—would strengthen the link between pharmacokinetics and clinical outcomes. Therapeutic drug monitoring in immunocompromised patients is also critical for optimizing treatment adjustments. Despite inherent limitations, well-designed real-world studies provide valuable insights not captured in randomized clinical trials, such as dose reductions, treatment interruptions, and unreported adverse effects [15]. Approaches such as propensity score matching (PSM) or inverse probability of treatment weighting (IPTW) further enhance the external validity of these studies [52,53].

### 4.6. Challenges in Oncology-Specific Evaluation

The maximum tolerated dose in healthy individuals does not necessarily correspond to that in cancer patients, due to disease-related alterations in organ function and pharmacokinetics [54]. Pharmacokinetic simulation studies highlight significant differences in absorption, distribution, metabolism, and elimination between healthy subjects and oncology patients, invalidating direct comparisons [55]. For example, liver cirrhosis in hepatocellular carcinoma significantly alters drug clearance [56]. Consequently, bioequivalence studies conducted in healthy volunteers provide insufficient evidence to determine safety and efficacy in oncological contexts.

### 4.7. Integrated Interpretation

Overall, the Delphi panel underscored the central importance of bioavailability and the need for rigorous clinical evaluation in the adoption of generic oncology drugs. These findings reflect structured expert consensus, highlighting concerns that even subtle formulation differences may translate into meaningful clinical consequences. Peer-reviewed studies reinforce these concerns by providing empirical evidence that generics may underperform relative to innovator products across both efficacy and safety endpoints. This convergence of expert opinion and published evidence demonstrates a consistent pattern: generics in oncology may compromise short-term outcomes such as biomarker control, response rates, and toxicity profiles, with downstream implications for long-term endpoints, including PFS and OS. While Delphi ratings capture clinician perceptions of risk and priority, case-based evidence illustrates these risks in practice, reinforcing the call for more stringent bioequivalence assessments and real-world monitoring.

Balanced Interpretation: Safety and Equity Considerations

While the Delphi panel and peer-reviewed studies highlighted substantial safety concerns, it is equally important to recognize the potential benefits of generics in oncology. Their lower cost can significantly improve equity of access, particularly in low- and middle-income countries, where high drug prices are one of the primary barriers to cancer care [3,12,57]. Broader uptake of generics may enable health systems to reach more patients, reduce catastrophic out-of-pocket expenditures, and reallocate limited resources toward newer and innovative therapies. From a sustainability perspective, the introduction of generics supports the long-term financial viability of oncology programs [58].

The Latin American context illustrates both opportunities and vulnerabilities. Agencies such as INVIMA face challenges ensuring rigorous oversight, given resource constraints and fragmented health systems. This can result in heterogeneous adoption of generics and inconsistent post-marketing surveillance. These limitations underscore the need for greater regional harmonization and capacity building, particularly in safety monitoring and regulatory review. By contrast, global regulatory agencies such as the U.S. Food and Drug Administration (FDA) and the European Medicines Agency (EMA) provide examples of more mature frameworks. The FDA’s Sentinel Initiative [59] is a large electronic system designed for active post-marketing surveillance of medical products, leveraging distributed data sources to identify safety issues in real time. Similarly, in 2010 the EMA adopted its revised Guideline on the Investigation of Bioequivalence (CPMP/EWP/QWP/1401/98 Rev.1) [60], setting standards for design, conduct, and evaluation of bioequivalence studies including considerations for highly variable drugs, narrow therapeutic index drugs, and use of scaling methods.

### 4.8. Limitations of Current Bioequivalence Studies

Current bioequivalence studies frequently fail to account for interindividual variability, which can produce false-negative results and mask clinically relevant differences. To address these limitations, regulatory frameworks must enforce high-quality study designs. The EMA’s 2010 guidelines sought to standardize the design, execution, and evaluation of bioequivalence studies [19]; however, challenges remain. Bioavailability is not only determined by the concentration of the active ingredient but also by its temporal variability within the body. Point measurements of variability lack statistical robustness and are unreliable indicators of therapeutic efficacy [61,62]. Moreover, the statistical power of bioequivalence studies is highly dependent on sample size and population variability, necessitating larger and more representative study designs [63].

### 4.9. Study Limitations

This study has several limitations. The Delphi consensus was based on a relatively small expert panel (19 participants), which may limit the generalizability of its recommendations, and the selection of experts—guided by professional experience rather than representative sampling—may have introduced selection bias. In addition, the regional focus on Latin America restricts the broader applicability of the findings, as regulatory frameworks and clinical practices differ globally. Finally, biosimilars were excluded by design, given their unique regulatory and clinical considerations; future work should address this important category in a dedicated analysis.

### 4.10. Priority Areas for Future Research

Future research should expand the scope by including larger and more diverse expert panels, while also conducting cross-regional comparisons to strengthen the global applicability of findings. Comparative clinical studies in oncology are needed to validate the efficacy and safety of generics in real-world settings, where treatment complexity and variability may not be fully captured in randomized trials. Integrating real-world evidence with prospective studies will provide a more comprehensive view of therapeutic performance. The development of pharmacokinetic and pharmacodynamic biomarkers of equivalence is a priority to enable more precise measurement of therapeutic comparability. In addition, efforts toward international regulatory harmonization—building on frameworks such as those of the FDA and EMA—will be critical to ensure consistency in approval standards and post-marketing surveillance across regions.

Finally, biosimilars, although excluded from this study, warrant dedicated evaluations given their increasing role in oncology. Focused research should address their unique regulatory, clinical, and economic considerations to complement the evidence base for generics.

## 5. Conclusions

This study highlights both the promise and the risks of generic oncology medications. The Delphi panel underscored bioavailability and clinical evaluation as central concerns, reflecting apprehension that even subtle formulation differences can have meaningful clinical consequences. Evidence from peer-reviewed studies reinforces these concerns, documenting reduced efficacy and increased toxicity with certain generics.

At the same time, generics represent an essential mechanism to expand access, reduce catastrophic expenditures, and sustain oncology programs—particularly in low- and middle-income countries where high costs remain a primary barrier to treatment. Realizing these benefits requires a balance: regulatory frameworks must enforce oncology-specific bioequivalence standards, strengthen pharmacovigilance, and promote international harmonization, while physicians must play an active role in formulary decisions and therapeutic monitoring.

Future work should broaden the evidence base by integrating real-world data, developing oncology-specific pharmacokinetic markers, and conducting comparative studies across regions. Biosimilars, although excluded here, warrant dedicated evaluation given their increasing relevance. Ultimately, aligning regulatory rigor with affordability will be critical to achieving equitable, safe, and sustainable cancer care.

## Figures and Tables

**Table 1:** Summary of Voting Scores on Generic vs. Innovator Drugs in Oncology.

Phase/ Domain	Key Finding	Recommendation	Reported Score (Mean/Median)
Open-Ended Questions (Phase I)	Innovator profile rated as predictable and manageable		Mean: 9.3
	Generic profile rated as predictable and manageable		Mean: 4.3
	Relevance of bioavailability in oncology		Mean: 9.8
Recommendations (Phase II)	Differences in bioavailability and impact on clinical efficacy	It is recommended to acknowledge that differences in bioavailability between innovator drugs and their generic versions can significantly affect clinical efficacy, particularly in oncology. Factors such as particle size, chemical polymorphisms, excipients used in generic formulations, and the quality of storage and the supply chain can influence drug absorption and, consequently, therapeutic effectiveness.	Mean: 9.9
	Evaluation of bioequivalence in oncology	It is recommended that comparative studies between innovator and generic oncology medications include robust non-inferiority trials that account for interindividual variability and assess clinically relevant outcomes, such as tumor response, survival, and toxicity. These studies would allow for more reliable comparisons within the oncology setting.	Mean: 9.9
	Assessment of bioequivalence in healthy volunteers and applicability to oncology	Assessing the bioequivalence of generic drugs through studies in healthy volunteers and small patient cohorts is insufficient to extrapolate findings to oncology patients. Cancer patients often exhibit altered pharmacokinetics due to changes in organ function—particularly in the liver, kidneys, and gastrointestinal tract—which can impact absorption, distribution, metabolism, and elimination [22,37].	Median: 9.9
	Differences in clinical outcomes between innovator and generic products	In clinical practice, significant differences in outcomes have been observed between patients treated with innovator products and those receiving generics, particularly in terms of therapeutic failures in oncology. Variability in toxicity and side effects has also been noted. These differences must be carefully considered in clinical decision-making.	Mean: 9.9
	Clinical outcomes to be evaluated in oncology (RR, QoL, PFS, OS)	From a clinical perspective, both short- and long-term outcomes should be evaluated to determine the efficacy and safety of cancer treatments. Short-term outcomes include response rate (RR), quality of life metrics, and toxicity profiles, which allow for early assessment of therapeutic benefit. In the long term, progression-free survival (PFS) and overall survival (OS) are key endpoints.	Mean: 9.8
	Importance of real-world studies in oncology	Real-life studies are becoming increasingly relevant in clinical practice, as they provide data that reflect situations closer to the everyday conditions of patients. These studies allow for the analysis of patient profiles and outcomes in scenarios that are more representative of the usual clinical environment, as opposed to controlled and randomized clinical trials, which tend to be more structured and select more homogeneous populations. However, although real-life studies are valuable, their level of evidence and design can still be questioned, especially when conducted with small patient samples (e.g., 30 or 100), which could generate doubts about the generalization of results.	Not reported

**Table 2:** Clinical Outcome Differences Between Innovator and Generic Oncology Drugs, Stratified by Cancer Type and End-points.

Cancer Type	Drug/Technology	Observed Differences with Generic	Oncology Endpoints Affected
Chronic Myeloid Leukemia (CML)	Imatinib	Higher rates of therapeutic failure [38,39]; increased adverse events [40,41], and toxicity [42,43]	Response Rate (RR), Progression-Free Survival (PFS), Overall Survival (OS), Toxicity
Prostate Cancer	Aromatase inhibitors	Increased PSA levels after switching to generics	Biochemical marker (PSA), Treatment Response, PFS
	Abiraterone	Reduced PSA suppression with generics	PSA response, Biochemical PFS
Multiple Solid Tumors	Docetaxel (generics)	Variable API content [34]; contamination (1–3%) [21]; increased febrile neutropenia [45].	Toxicity profile, QoL, Treatment Adherence, PFS/OS (indirect)
	Cisplatin	Higher renal failure rates (21% vs. 9%, *p* < 0.001) [44].	Toxicity (renal), QoL, Treatment Continuity, OS (indirect)

## Data Availability

The data used in this consensus development process, including clinical findings, literature review, and discussions from the expert panel, are available upon reasonable request to the corresponding author.

## Supplementary Materials

The following supporting information can be downloaded at: https://www.mdpi.com/article/10.3390/jcm14217543/s1, Table S1: Regulation and definitions of generic medications in various Latin American countries [13,14,15,64].

## Abbreviations

The following abbreviations are used in this manuscript:

ANMAT	Administración Nacional de Medicamentos, Alimentos y Tecnología Médica (Argentina)
API	Active Pharmaceutical Ingredient
ASD	Amorphous Solid Dispersion
BCS	Biopharmaceutical Classification System
CHMP	Committee for Medicinal Products for Human Use
CI	Confidence Interval
CML	Chronic Myeloid Leukemia
COFEPRIS	Comisión Federal para la Protección contra Riesgos Sanitarios (Mexico)
DCI	Denominación Común Internacional (International Nonproprietary Name)
DIGEMID	Dirección General de Medicamentos, Insumos y Drogas (Peru)
EMA	European Medicines Agency
FDA	Food and Drug Administration (United States)
GI	Gastrointestinal
GMP	Good Manufacturing Practices
ICH	International Council for Harmonisation of Technical Requirements for Pharmaceuticals for Human Use
INVIMA	Instituto Nacional de Vigilancia de Medicamentos y Alimentos (Colombia)
IPTW	Inverse Probability of Treatment Weighting
ISP	Instituto de Salud Pública (Chile)
MGI	Medicamento Genérico Intercambiable (Interchangeable Generic Drug)
OS	Overall Survival
PFS	Progression-Free Survival
PSA	Prostate-Specific Antigen
PSM	Propensity Score Matching
RR	Response Rate
SORDOS	Systematize, Organize, Respond, Disseminate, Advocate, Synthesize
U.S.	United States
WHO	World Health Organization
