# Supplementary material for: Optimization of the Use of Generic Medications in Oncology: Improving Safety and Therapeutic Quality
Source: J Clin Med. 2025 Oct 24;14(21):7543. doi: 10.3390/jcm14217543 (PMC12610037; doi:10.3390/jcm14217543)
Supplement: Supplementary file 1 [file jcm-14-07543-s001.zip › jcm-3838113-supplementary.pdf]

Supplementary Materials: Supplement A.

Table S1. Regulation and definition of generic medications in various Latin American countries [13–15,64].

Country	Regulation	Regulatory Authority	Definition	Requirements
**Argentina**	Law 25.649 of 2002	National Administration of Medicines, Food and Medical Technology (ANMAT)	Similar Drug: A product with the same active ingredients, pharmaceutical form, route of administration, and therapeutic indications as the reference drug. It may differ in characteristics such as size, shape, excipients, and primary packaging but must demonstrate quality, safety, and efficacy.	All drugs require equivalence studies. The type of test is defined according to the drug category. Bioequivalence tests are regulated by ANMAT provision 5040/06.
**Bolivia**	Drug Law of 1996 No. 5040	National Unique Supply System and the National Drug Surveillance and Control System	Multi-source pharmaceutical product from more than one manufacturer, equivalent or alternative, which may or may not be therapeutically equivalent.	Requires bioavailability and bioequivalence studies with technical documentation proof.
**Brazil**	Law No. 9787 of 1999	National Health Surveillance Agency (ANVISA)	Generic drug: A product similar to a reference or innovator drug, with demonstrated efficacy, safety, and quality. It is interchangeable with the original and generally produced after the expiration or renunciation of the patent. It is designated with the Brazilian common name or, in its absence, with the International Common Name (DCI).	Generic drugs must be pharmaceutical and therapeutic equivalents to the innovator molecule. In vitro tests are conducted by national laboratories authorized by ANVISA. Therapeutic equivalence is verified through in vivo tests.

**Chile**	Resolution 244-ISP Chile	Public Health Institute of Chile (ISP)	Multi-source pharmaceutical product that may be therapeutically equivalent or not to the pharmaceutical product that serves as a reference.	Bioequivalence studies are required for certain generic drugs. In 2005, with resolution Ex. 727, the norm defining the criteria for establishing therapeutic equivalence in pharmaceutical products was approved, with a list of active ingredients requiring in vivo or in vitro equivalence.
**Colombia**	Decree Number 677 of 1995 and 1505 of 2014. Resolution 662 of 2022, 1124 of 2016, and 1400 of 2001	National Institute for Food and Drug Surveillance (INVIMA)	The regulation states that a generic drug is a pharmaceutical product containing an active ingredient recognized in the Colombian Pharmacological Standards. It differs from an innovator in that it has not been fully developed from its chemical synthesis to its clinical use.	Resolution 1124 of 2016 establishes the guide for the bioavailability and bioequivalence of drugs. Since 2001, the forms and pharmaceutical groups that must present bioavailability and bioequivalence studies were defined.
**Costa Rica**	Not specified	Ministry of Health	Multi-source pharmaceutical product: Pharmaceutical equivalent that may or may not be therapeutically equivalent. Generic drug: Marketed under the common name of the active ingredient without a brand name.	Bioequivalence studies are required for drug registration. In 2007, a list of active ingredients of drugs that must comply with bioavailability and bioequivalence tests was established.
**Mexico**	Not specified	Federal Commission for the Protection against Sanitary Risks (COFEPRIS)	Interchangeable Generic Drug (MGI): Product with same active ingredient, concentration, dosage form, and route as the reference drug. Must demonstrate	Must comply with Good Manufacturing Practices (GMP) and meet dissolution and bioequivalence tests. COFEPRIS authorizes laboratories to conduct the required

			bioequivalence and be listed in the MGI catalog.	studies. Since 2008, all drugs must be evaluated for a period of two years to demonstrate efficacy, safety, and quality.
**Panama**	Law 1 of January 10, 2001, and Decree No. 6 of February 21, 2005	National Directorate of Pharmacy and Drugs of the Ministry of Health	Multi-source drug that may be interchangeable with the reference product, typically produced post-patent without innovator license.	Manufacturing laboratories or representatives must present evidence of therapeutic equivalence through pharmacodynamic studies, bioequivalence, clinical, or comparative dissolution profile studies, depending on the type of drug.
**Peru**	Decree 024-2018-SA and resolution 404-2021/MINSA	General Directorate of Medicines, Supplies, and Drugs (DIGEMID)	Interchangeable drug: A drug that has the same active ingredient, dosage, pharmaceutical form, and route of administration as an innovator drug.	A generic drug must comply with bioequivalence and physicochemical studies.
**Uruguay**	Not specified	Ministry of Public Health (MSP)	Interchangeable Drug: A similar drug or pharmaceutical alternative that has demonstrated biopharmaceutical equivalence with the reference drug through established procedures. Similar or Pharmaceutical Equivalent Drug: Contains the same active ingredient, concentration, and pharmaceutical form as the original but may	There are drugs that, for safety reasons, cannot be registered as generic drugs.

			differ in size, shape, excipients, packaging, and labeling.	
**Guatemala**	Decree Number 90-97, Law on Medicines and Related Products	Ministry of Public Health and Social Assistance (MSPAS) and Directorate for Health Regulation, Surveillance and Control (DRVyCS)	Generic medicine: a pharmaceutical product that is therapeutically equivalent to a reference medicine, with the same active ingredient, concentration, pharmaceutical form, route of administration, and bioavailability.	Bioequivalence studies are required for essential medicines and those with a narrow therapeutic index. Good Manufacturing Practices are mandatory for all.
